# Supplementary figures and images for: Development of a CYP2D6-enhanced HepaRG cell model with improved CYP2D6 metabolic capacity
Source: PLoS One. 2025 Dec 29;20(12):e0339559. doi: 10.1371/journal.pone.0339559 (PMC12747322; doi:10.1371/journal.pone.0339559)

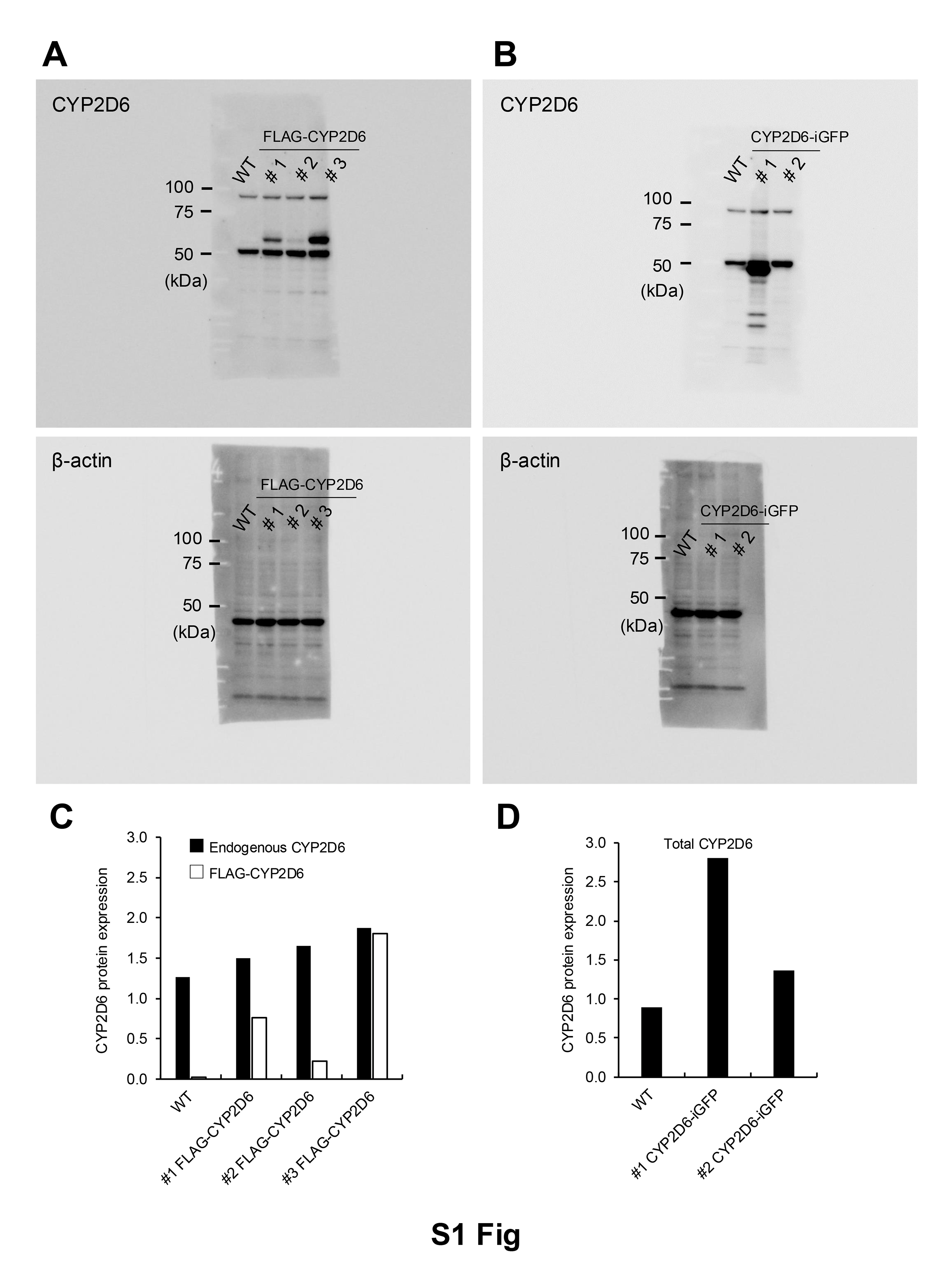

Supplement: S1 Fig — (A and B) Uncropped raw images of Western blotting analysis related to Figure 1D (A) and Figure 2D (B). (C and D) Quantification of CYP2D6 expression normalized by b-actin in FLAG-CYP2D6 cell lines (C) and CYP2D6-iGFP cell lines (D). (TIF) [file pone.0339559.s001.tif]

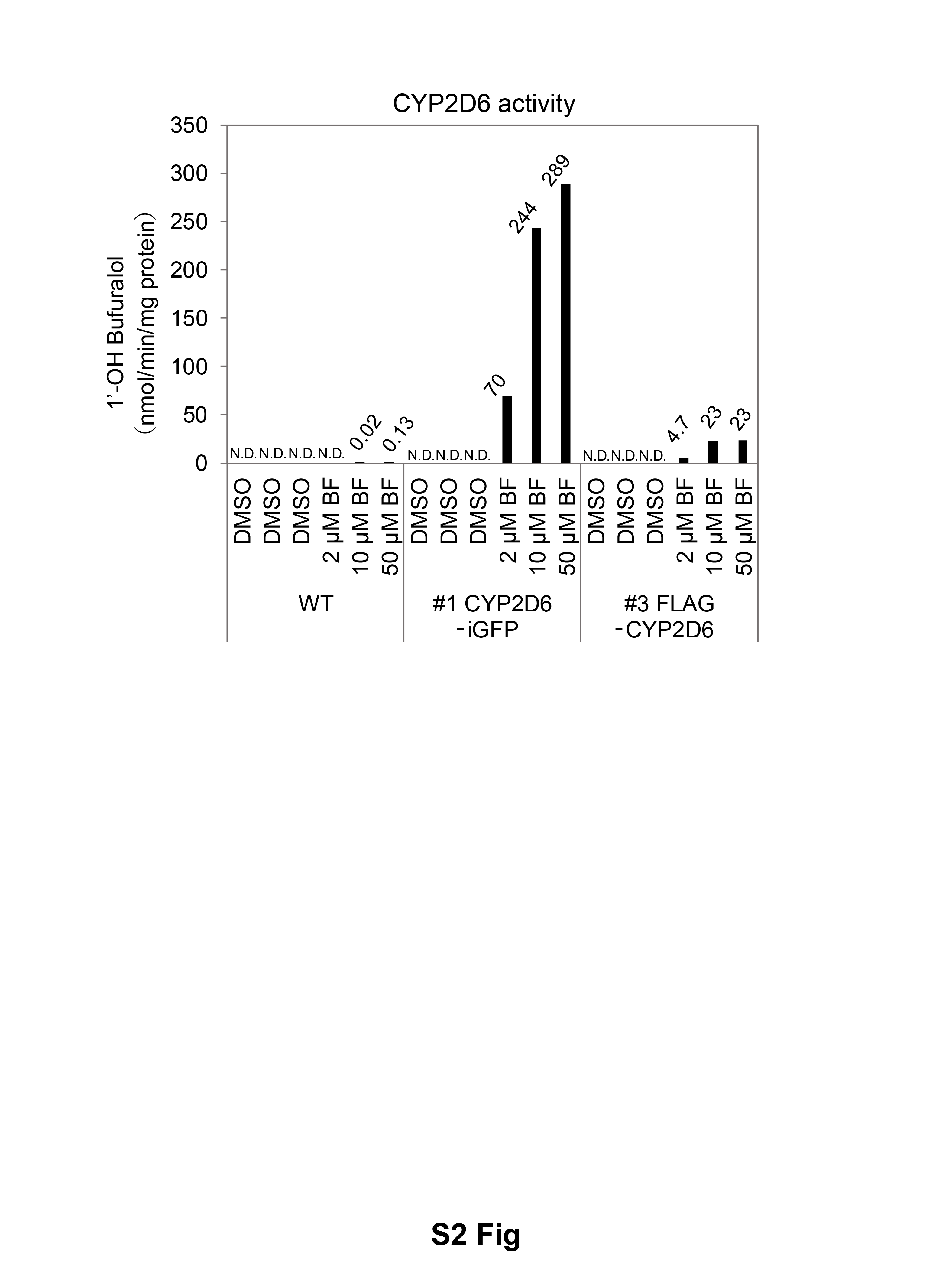

Supplement: S2 Fig — HepaRG cells were incubated with varying concentrations of bufuralol for 24 h. The amount of 1’-OH bufuralol produced was measured using LC-MS/MS analysis. BF indicates the bufuralol-treated group. DMSO was used as a vehicle control without a substrate. (TIFF) [file pone.0339559.s002.tiff]

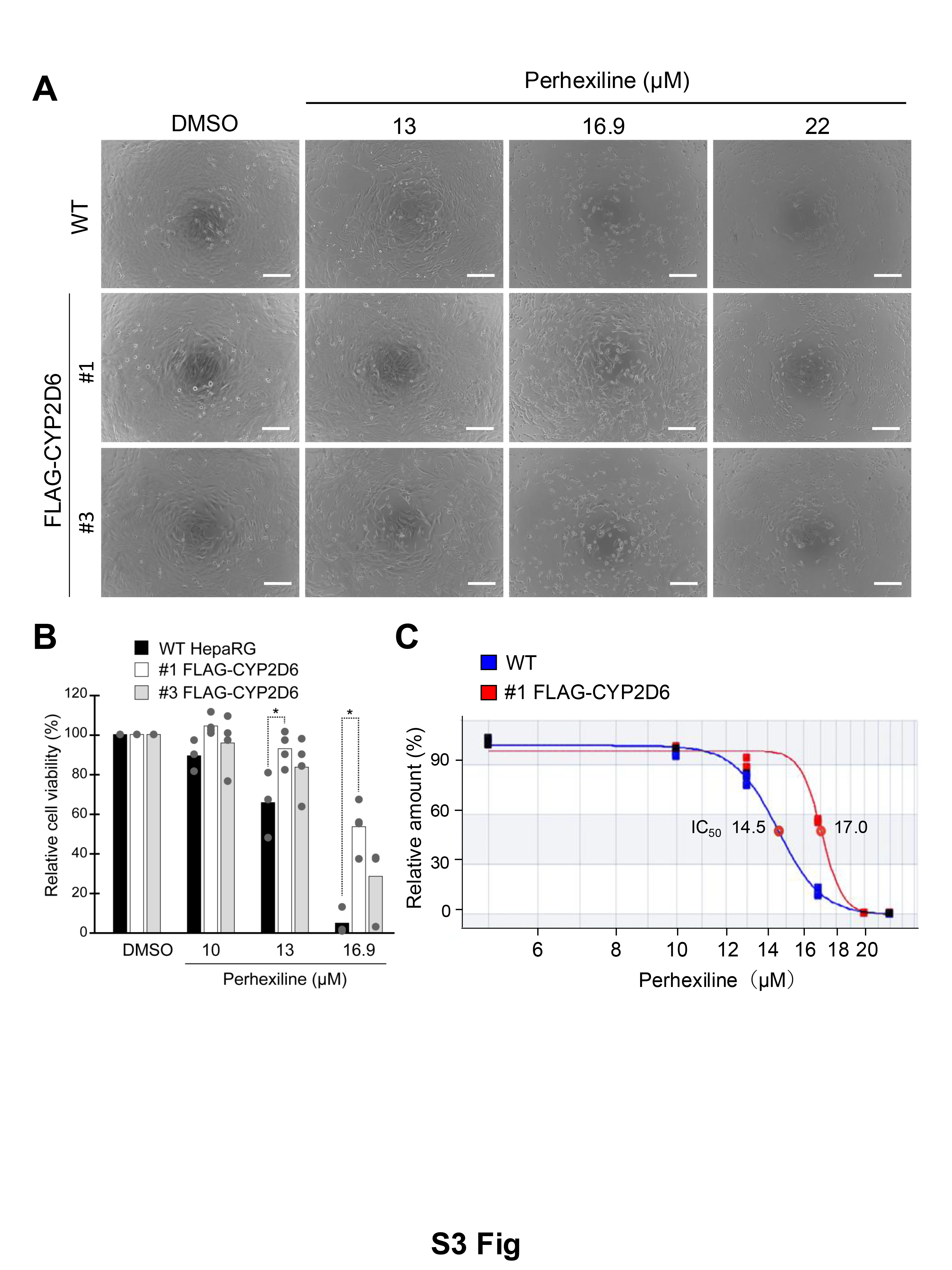

Supplement: S3 Fig — (A) Representative images of WT and FLAG-CYP2D6 HepaRG cells (#1 and #3) after 24-hour exposure to perhexiline. Phase-contrast microphotographs are shown. Scale bar, 200 µm. (B) Relative cell viability of perhexiline-treated cells assessed by an ATP-based cell viability assay. Each dot represents an individual replicate (n = 3–4). P value calculated from Dunnett’s multiple comparison test. *P < 0.05. (C) Dose-response curves of relative cell viability in perhexiline-treated cells. Each dot represents an individual value. The estimated IC50 value is indicated by a red closed circle. n = 3. (TIFF) [file pone.0339559.s003.tiff]

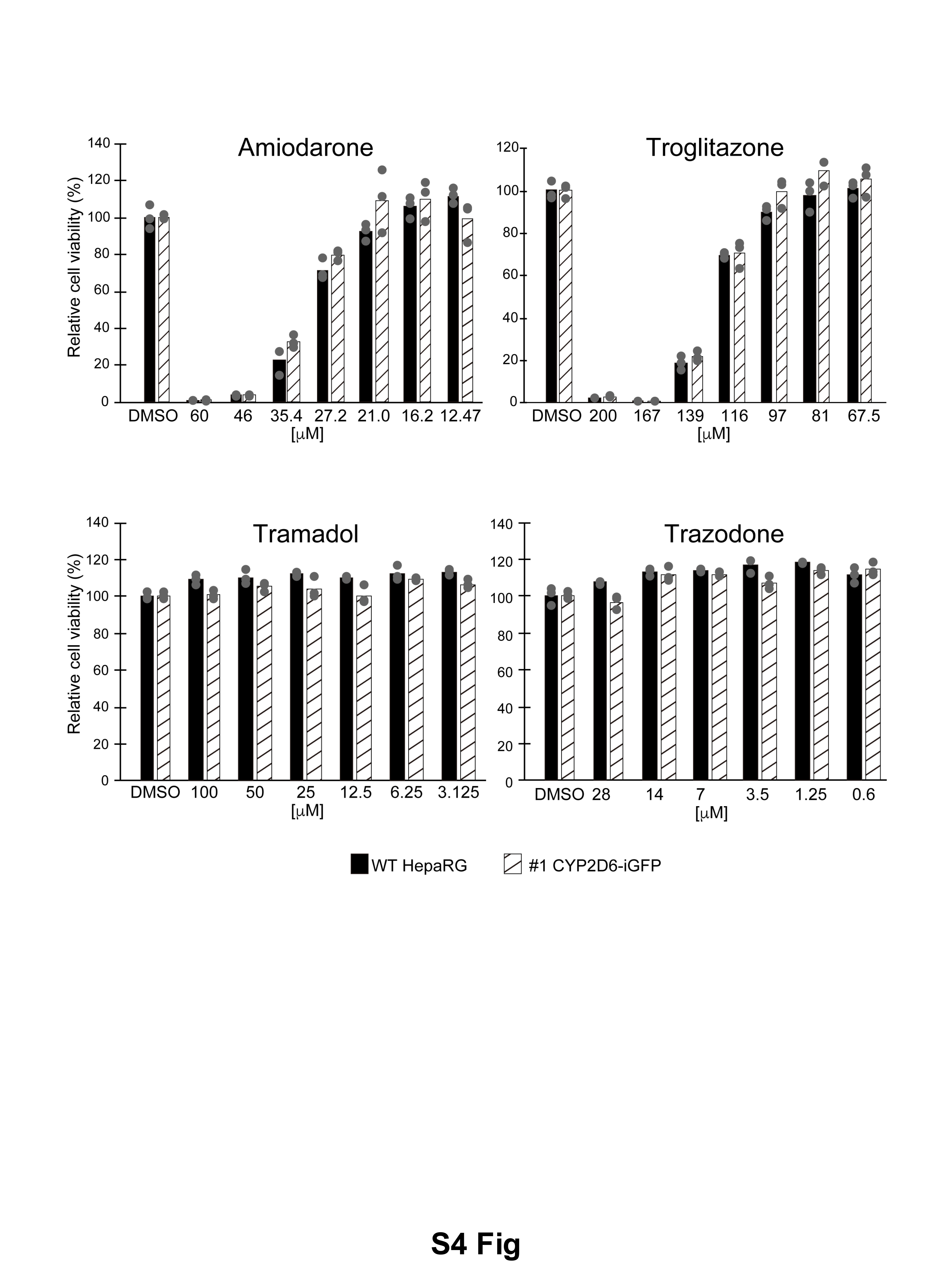

Supplement: S4 Fig — Relative viability of cells treated with known DILI-related compounds. Error bars, SD. n = 3. (TIFF) [file pone.0339559.s004.tiff]

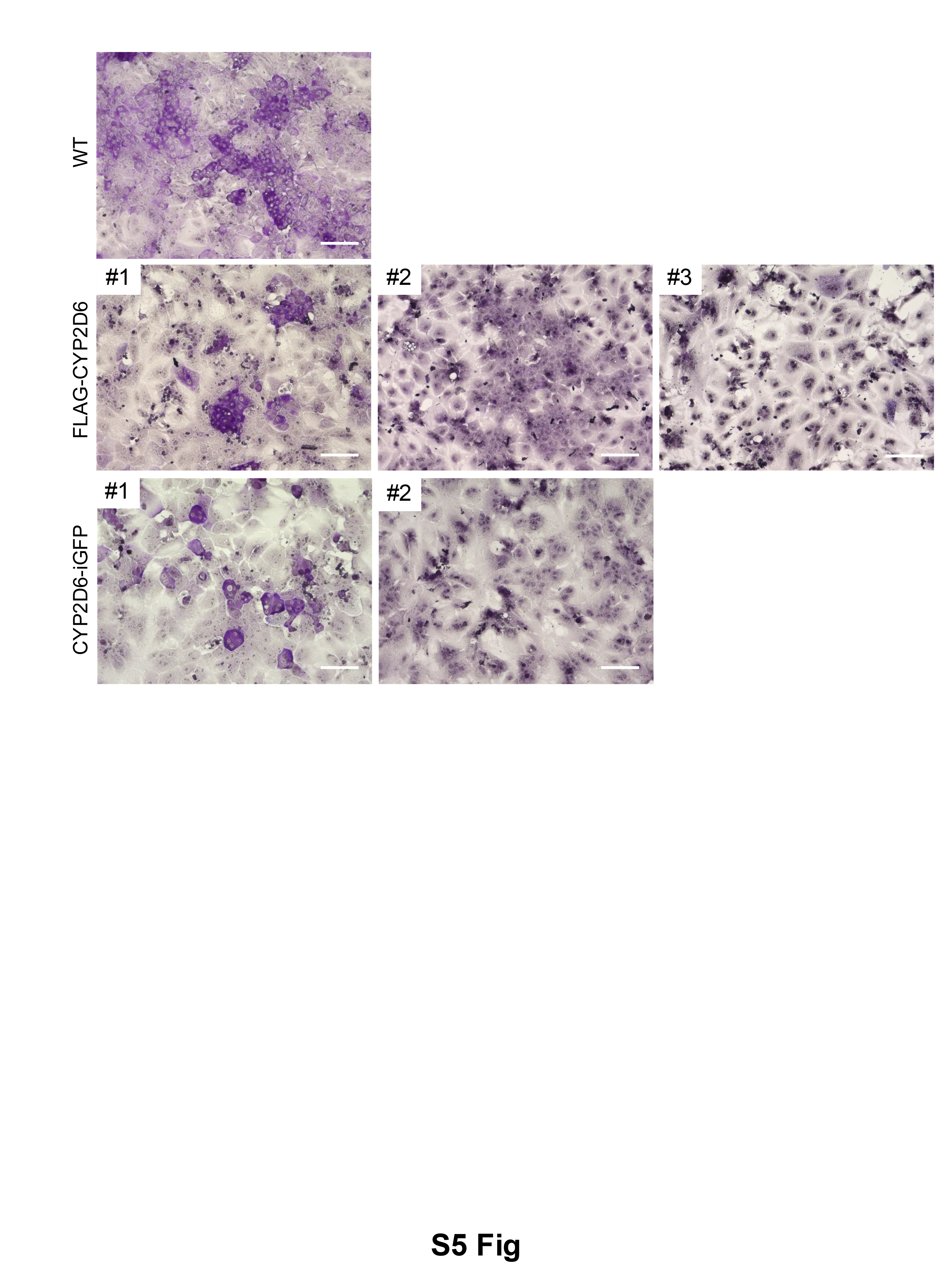

Supplement: S5 Fig — (A) Representative images of PAS-stained cells in WT, FLAG-CYP2D6 and CYP2D6-iGFP HepaRG cell lines. PAS staining was performed at 28-day differentiation. Scale bar, 100 µm. (B) Representative images of PAS-stained cells in WT, #1 FLAG-CYP2D6, and #1 CYP2D6-iGFP HepaRG cells. After hepatic differentiation, PAS staining was conducted. Scale bar, 100 µm. (TIFF) [file pone.0339559.s005.tiff]

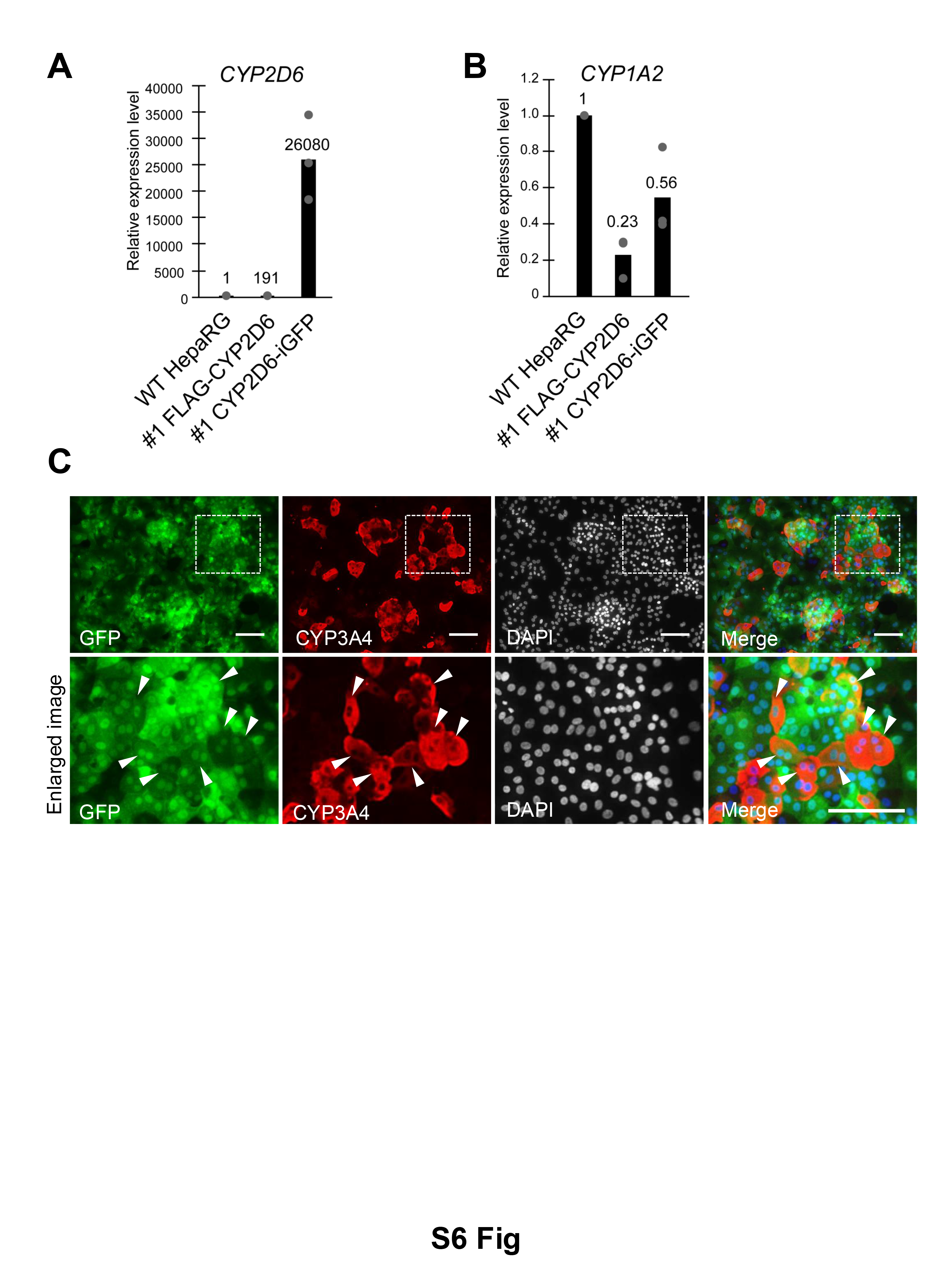

Supplement: S6 Fig — The gene expression levels of (A) CYP2D6 and (B) CYP1A2 in differentiated cells. Each dot represents an individual replicate (n = 3). (C) Representative immunofluorescence images showing the CYP3A4 expression in the differentiated #1 CYP2D6-iGFP cells. The enlarged area is outlined by the dotted square. Arrowheads indicate double-positive cells for CYP3A4 and GFP in differentiated #1 CYP2D6-iGFP cells. Scale bar, 100 µm. (TIF) [file pone.0339559.s006.tif]
