# Supplementary material for: Development of a CYP2D6-enhanced HepaRG cell model with improved CYP2D6 metabolic capacity
Source: PLoS One. 2025 Dec 29;20(12):e0339559. doi: 10.1371/journal.pone.0339559 (PMC12747322; doi:10.1371/journal.pone.0339559)
Supplement: Attachment — Submitted filename: Response to Reviewers.docx [file pone.0339559.s007.docx]

**Response to Editor and Reviewers**

**Manuscript ID:** PONE-D-25-31242
**Title:** *Development of a CYP2D6-enhanced HepaRG Cell Model with Improved CYP2D6 Metabolic Capacity*

We thank the Academic Editor and the Reviewers for their constructive comments. We have revised the manuscript accordingly. Below we respond to each point and indicate where changes were made in the revised files.

**A. Responses to Journal Requirements**

**1. Please ensure that your manuscript meets PLOS ONE's style requirements, including those for file naming.**
**Response:** We reformatted the manuscript to comply with PLOS ONE templates (title page, main text structure, figure legends, references, file names).

**2. Please state what role the funders took in the study.**
**Response:** We added the following statement to the cover letter and Funding section:
*“The funders had no role in study design, data collection and analysis, decision to publish, or preparation of the manuscript.”*

**3. “Data not shown” not permitted**
**Response:** All instances of “data not shown” were removed.

**4. Ethics statement in Methods**
**Response:** During our internal compliance check, we recognized that the diastase control used for PAS staining had been generated with fresh human saliva from one of the authors as an amylase source. Under our institutional policy and PLOS ONE’s ethics requirements, the use of human-derived material requires an IRB approval or an official exemption determination. Because retrospective determinations are generally not feasible and to avoid any ambiguity, we removed this dataset entirely (previous Figure S5B). The diastase-treated panel served only as an auxiliary confirmation for PAS staining and **does not affect any analyses or the conclusions regarding enhanced CYP2D6 metabolic capacity**. We have deleted all references to “diastase” from the Methods/legends and updated the figures accordingly.

**5. Uncropped raw images for blots/gels**
**Response:** We provide original, uncropped, unadjusted images for all blots as **S1 Fig** and reference them in figure legends.

**6. Optional citation additions suggested by reviewers**
**Response:** We evaluated suggested literature domains (HepaRG protocols, CYP2D6 in HepaRG, regulatory guidance context) and added relevant citations where appropriate (Line 112-113, reference 12, 21, and 22).

**B. Responses to Additional Editor Comments**

**1. DMSO schedule (1% for 2 days → 1.7% for 12 days): standard or optimized? Add reference.**
**Response:** We added relevant citations (Line 112-113, reference 12, 21, and 22).

**2. “Consistent with previous reports, CYP2D6 catalytic activity is low in WT HepaRG despite detectable protein by western blot”—add references.**
**Response:** We added relevant citations (Line 315, reference 15, 26, and 27).

**3. Consider PMDA guidelines in comparison to the FDA Modernization Act 2.0; consider other regulatory bodies.**
**Response:** We appreciate this suggestion. In the Discussion, we added a brief paragraph positioning our model within the policies of the Pharmaceuticals and Medical Devices Agency (PMDA) and the European Medicines Agency (EMA) regarding non-animal, human cell–based approaches, with appropriate citations (Line 436-441, reference 27).

**4. More detailed statistical analysis; add references; address R1/R2 points.**
**Response:** See Responses to Reviewers below.

**5. As per reviewer-1, additional experiment is suggested to carry out and support the work. As per reviewer-2, add appropriate abbreviations, figure legends, image quality enhancement.**
**Response:** We have addressed these points; details are provided in the point-by-point responses below.

**C. Responses to Reviewer #1**

We thank the reviewer for the careful evaluation and have clarified the strengths of our study where appropriate. We overlaid all individual measurements to enhance transparency of the descriptive statistics and corrected the noted line-spacing issue. Specific changes are described below.

**1. HepaRG control if possible empty vector control could also be included**
**Response:** We appreciate the suggestion and understand its rationale. Because our objective was to develop a CYP2D6-enhanced HepaRG platform, the appropriate negative comparator is the parental (WT) HepaRG line; adding an empty-vector control would mainly assess backbone effects and is unlikely to strengthen our platform-focused conclusions, so we did not add this experiment.

**2. The descriptive statistics part should be presented as additional data; the means with SD but it is also possible to include median and interquartile range if the data is skewed is better to be considered.**
**Response:** Thank you for this helpful suggestion. We revised the figures to overlay all individual measurements on each graph, so readers can directly assess the data distribution and variability (Fig 1F, H, I, Fig 2C, S4, S6 Fig A, B). These updates improve transparency and do not alter our conclusions.

**3. Assumption of normality and equal variance needs to be done and mentioned if it was done**

**Response:** We revised method section (Line246-247).

**4. Line 176,177,178 line spacing to be corrected.**
**Response:** We apologize for the line-spacing issue at lines 176–178; it has been corrected in the revised manuscript.

**D. Responses to Reviewer #2**

We are grateful for your thoughtful and practical suggestions on presentation. In response, we standardized abbreviations, revised all figure legends and image quality to match PLOS ONE format, moved Supplementary Tables 1–2 into the main text, and clarified how p values were calculated and which statistical model underlies our framework. Specific changes are described below.

**1) Abbreviate the short-terms used in manuscript.**
**Response:** We revised the manuscript to define all abbreviations at first mention and to use them consistently thereafter.

**2) Check out recent PlosOne publications and format your manuscript accordingly, In-text/ end bibliography, manuscript sections and sub-headings, also assign numbers to headings and their sub-headings.**
**Response:** We reformatted per PLOS ONE conventions; we adopted a numbered heading scheme consistently and standardized references and in-text citations.

**3) Kindly, arrange figure legends and resolution according to Journal's format.**
**Response:** We rearranged figure legends according to PLOS ONE's format.

**4) Include, Supplementary Table 1 and 2, into Main data.**
**Response:** We promoted **Supplementary Table 1/2** into the main text as **Table 1/2** (with concise summaries).

**5) How did you calculated p value, elaborate briefly.**
**Response:** We clarified that we report Dunnett-adjusted p values, with significance determined at α = 0.05 (two-sided). We revised method section (Line249-250).

**6) which statistical model is used to design this experimental framework.**
**Response:** We clarified that the framework is a fixed-effects one-factor linear model (group as the factor) with pre-specified Dunnett contrasts comparing each clone to the common WT control. Tests are two-sided with α = 0.05, and we report Dunnett-adjusted p values; the unit of analysis is biological replicates (technical replicates averaged). We revised method section (Line247-250).
